# No escape: crisis of extreme heat indoors

**DOI:** 10.1016/j.lansea.2026.100799

**Published:** 2026-06-11

**Authors:** The Lancet Regional Health – South East Asia

Each year as summer arrives, we are inundated by hourly updates on heat maps, forecasting extreme heat days with advisories from local authorities to stay indoors and hydrated. On Heat Action Day 2026, however, we must confront an uncomfortable truth: for countries in south Asia, staying indoors is no longer a reliable message as the dangers of extreme heat now also follow us inside.

The Lancet Countdown estimated that global heat-related mortality has risen by 23% since the 1990s, reaching approximately **546,000 deaths annually worldwide** between 2012 and 2021. A recent modelling study using Global Burden of Disease data projected that heat-attributable deaths could exceed **400,000 annually by 2045** if warming continues, with the highest risk predicted for countries in south Asia. These numbers could be a gross underestimate as challenges persist in recording heat-related deaths, especially in the presence of comorbidities such as cardiovascular disease, stroke, or kidney disease. Moreover, due to a lack of high-quality data on its direct impact on health, indoor heat in particular remains insufficiently addressed in current policy frameworks.

Countries in south Asia have been experiencing longer, earlier, and more intense heatwaves in the recent past, with rising night-time temperatures. For millions of people living in densely populated urban settlements, limited access to cooling, lack of basic amenities such as electricity, water, and ventilated housing with thermally safe construction, and the presence of dangerous air pollution, make it difficult to maintain hydration, hygiene, and comfort during prolonged hot periods. Cool roofs, shading, ventilation, low-heat-gain materials, and urban greening can reduce indoor temperatures without increasing electricity demand. Encouragingly, parts of south Asia have already demonstrated that such initiatives can provide relief during peak season with much coordinated action. In India, Ahmedabad’s Heat Action Plan—developed after the devastating 2010 heatwave—integrated early warning systems, health-care preparedness, public communication, and later low-cost cooling interventions such as cool roofs for low-income settlements. Similar initiatives in schools and for vulnerable communities in Tamil Nadu and Maharashtra suggest that passive cooling and community-based adaptation can reduce indoor heat exposure without deepening dependence on energy-intensive air-conditioning. India is one of the first countries with a national cooling framework, the India Cooling Action Plan (ICAP). Yet even these examples reveal persistent inequities in implementation, access, and long-term urban planning.

Heat adaptation policies in south Asia remain alarmingly incomplete because they focus on outdoor temperatures while neglecting indoor heat. Current housing schemes often prioritise quantity over thermal safety. Infrastructure adaptation with a special focus on women, older people, and children requires urgent policy attention. Labour and occupational policies in the region are underdeveloped in the wake of growing heat crisis. These need to include robust standards for factories, kitchens, warehouses, garment units, informal workshops, and domestic workers. Primary care and emergency systems should be equipped to screen for heat signs and handle any emergencies. Most heat-health surveillance systems today track outdoor temperatures, not indoor exposure. Therefore, local governance should prioritise indoor temperature monitoring, ward-level heat mapping, and identification of high-risk households.

Broad climate change policies take a long time to implement and influence change at a local level. In these circumstances, involving decentralised governance and empowering communities to implement local adaptive solutions might be more pragmatic and sustainable. Several initiatives across countries in south Asia suggest that bottom-up approach can be feasible and socially acceptable. In parts of India, community-led cool-roof programmes and women-led housing adaptations have improved indoor thermal comfort. Gram panchayats in Vidarbha and Chhattisgarh in India are beginning to integrate water conservation, heat mitigation, and climate adaptation into local development planning. Bangladesh has integrated local adaptation and social protection through community disaster-management structures. In many south Asian communities, resilience to heat is already practiced daily through informal adaptation, shared resources, and traditional knowledge. However, the challenge is to support, protect, and scale these practices.

On Heat Action Day 2026, the focus on indoor heat therefore represents more than a seasonal concern. Indoor heat must now be recognised not merely as a discomfort, but as a preventable environmental and occupational health risk that reflects the interconnected crises of climate change, water insecurity, pollution, and social inequity. Protecting populations from extreme heat will therefore require not only emergency advisories, but sustained investments in heat-resilient housing, equitable cooling, water security, occupational protection, and climate-sensitive public health systems implemented through strong local governance.

